# Evaluation of 563 children with chronic cough accompanied by a new clinical algorithm

**DOI:** 10.1186/s13052-015-0180-0

**Published:** 2015-10-06

**Authors:** Ahmet Hakan Gedik, Erkan Cakir, Emel Torun, Aysegul Dogan Demir, Mehmet Kucukkoc, Ufuk Erenberk, Selcuk Uzuner, Mustafa Nursoy, Emin Ozkaya, Fadlullah Aksoy, Selim Gokce, Kayhan Bahali

**Affiliations:** Division of Pediatric Pulmonology, Faculty of Medicine, Bezmialem Vakif University, Adnan Menderes Bulvarı (Vatan Cad.) Fatih, Istanbul, 34093 Turkey; Pediatrics, Bezmialem Vakif University, Istanbul, Turkey; Pediatric Allergy, Bezmialem Vakif University, Istanbul, Turkey; Otorhinolaringology, Bezmialem Vakif University, Istanbul, Turkey; Pediatric Gastroenterelogy, Bezmialem Vakif University, Istanbul, Turkey; Department of Child and Adolescent Psychiatry, Bakirkoy Research and Training Hospital for Psychiatry, Neurology and Neurosurgery, Istanbul, Turkey

**Keywords:** Chronic cough, Child, Cough algorithm

## Abstract

**Background:**

This study aims to evaluate the children with chronic cough and to analyze their etiological factors according to the age groups.

**Method:**

Five hundred sixty-three children with chronic cough were included. The last diagnosis were established and were also emphasized according to the age groups.

**Results:**

The mean age was 5.4 ± 3.8 years (2-months–17-years) and 52 % of them were male. The most common final diagnosis from all the participants were: asthma (24.9 %), asthma-like symptoms (19 %), protracted bacterial bronchitis (PBB) (11.9 %), and upper airway cough syndrome (9.1 %). However, psychogenic cough was the second most common diagnosis in the subjects over 6 years of age.

**Conclusion:**

Asthma and asthma-like symptoms were the most common diagnosis in children. Different age groups in children may have a different order of frequencies. Psychogenic cough should be thought of in the common causes especially in older children.

## Background

The chronic cough has been variably defined as a cough lasting longer than 4 weeks in children [1]. The personal burden of this symptom includes an impaired quality of life, multiple doctor visits, absences from school, and excessive medication expenses [2].

Different guidelines and clinical algorithms are used to evaluate the chronic cough [3–6]. A new, evidence-based, management pathway was made by Chang et al. in 2010, and was shown to be both feasible and reliable [7]. Managing children with chronic cough, in accordance with a standardized algorithm, showed an improvement in patient outcomes, and based on this good evidence, physicians might be more likely to alter their practice [1, 3].

The possible underlying etiologies of chronic cough are included in a very wide spectrum, ranging from a simple, non-specific cough, to a cough with more serious causes [8]. These causes can be divided into two different groups: specific and non-specific [7]. However, numerous specialists have conducted trials, which report that highly variable lists of etiologies are possible due to some factors [9]. Moreover, depending on the different ages in childhood, the most common causes may change [10].

Despite the importance of the chronic cough, data on the prevelance, predictors, etiology, and natural history of the symptoms are scarce [3]. In this study, we aimed to evaluate the children with chronic cough accompanied by the Chang et al. algorithm [7]. We also analyzed the characteristics, and etiological factors with regards to each age groups.

## Method

Patients with chronic cough, who were admitted to the Bezmialem Vakif University Hospital, Department of Pediatrics, Division of Pediatric Pulmonology, and Division of Pediatric Allergy clinic, were included in the study between October 2012-October 2013. Bezmialem Vakif University Hospital is one of the largest hospital in Istanbul, which provides secondary and tertiary services to a growing number of patients. The chronic cough is defined as a cough lasting longer than 4 weeks without being resolved during this period. The patients were evaluated by a cough algorithm written and published by Chang et al. in 2010 [7]. Permissions were obtained from the parents and ethical approval was taken from the local ethics committee of Bezmialem Vakif University.

### Inclusion and exclusion criteria

Our inclusion criteria was that the children had to be under 17 years of age, with a chronic cough and had to be admitted to our hospital for the first time. Exclusion criteria included children with a previously known, chronic respiratory illness showing cases of asthma, cystic fibrosis (CF), and bronchiectasis. The patients with neuromotor growth deficiency, cardiac or syndromic diseases, and premature birth were also excluded.

### Study design and patients

After the initial evaluations in different sections, the participants were referred to the Division of Pediatric Pulmonology. The demographical characteristics including age, gender, and birth date were obtained. The patients underwent a detailed medical history, which included information about the duration and the characteristics of the cough, and the presence of asthma-like symptoms. Exposure to passive smoking, atopy and asthma in the family, were looked at as well. A detailed physical examination was completed.

A cough diary was received from each participant. A cough diary is a validated scale, which is a verbal category, with a descriptive score ranging from 0 (no cough), to 5 (cannot do most usual activities due to a severe cough [11]. During each visit the cough diary was presented, and the results were noted, in order to evaluate the resolving of the cough for each participant.

The complete blood count, along with the posterior-anterior chest X-rays (CXR) were taken from all patients, while pulmonary function tests (PFT) were performed on patients who were able to cooperate. The following tests were performed on the appropriate patients: serum immunglobulin (Ig) levels, IgG subgroups, allergic skin test, specific IgE levels, sweat chloride test, tuberculin skin test, thorax high resolution computarized tomography (HRCT), flexible fiberoptic bronchoscopy (FFB) (Pentax, Hoya Corporation, Tokyo, Japan), bronchoalveolar lavage fluid (BALF) culture, esophageal Ph/impedence monitoring, gastroduodenoscopy, and echocardiographic examinations.

Lung function was measured by spirometry [Spirolab III (Medical International Research, Italy)], and interpreted by a pediatric pulmonologist. Lung volume were measured according to standard criteria [12]. Skin tests for spesific allergens were considered positive, if they produced a wheal response of at least 3 mm greater than the negative control after 15 min of application [13]. For the Ph study monitoring, if the ph in the distal esophagus was < 4 for > 5 % of the duration, the result was regarded as being abnormal [14].

Pediatric gastroenterologist evaluated patients with recurrent vomiting, with reflux symptoms associated with the upper airway tract, and with other gastrointestinal problems. An ear, nose and throat evaluation, including laryngoscopy, was performed by the otorhinolaringologist, in selected cases of those who had persistant upper airway disease symptoms. A pediatric psychiatrist examined the patients suspected of having a diagnosis of a psychogenic cough.

### Evaluation of the chronic cough

All participants were followed for at least 12 months. After the initial analyses on admission, the participants were evaluated according to the Chang et al. algorithm. All patients were examined for specific cough pointers (Table 1). The specific cough pathway was used for the patients who had these pointers, but there was a lack of these signs, the non-specific cough pathway was used [7]. Specific further tests were made and treatments were administered according to the cough pointers and primary diagnosis. Until the cough was resolved, and a concluding diagnosis was complete, these patients were repeatedly examined every 2–4 weeks, and followed-up with every 2 months thereafter. Their final diagnosis was also emphasized according to the age groups.

**Table 1:** Spesific cough pointers

Auscultatory abnormalities
Abnormal chest X-ray
Abnormal spirometry results
Wheezing
Dsypnea
Hemoptysis
Moist or productive coughs for more than 3 months
Chest pain
Classical cough characteristics
Recurrent pneumonia
Chest wall deformities
Feeding difficulties including vomitting
Digital clubbing
Immune deficiencies
Cardiac abnormalities
Neurodevelopmental abnormalities
Failure to thrieve

### Definitions of the specific causes

#### Asthma and reactive airway disease (asthma-like symptoms)

The asthma diagnosis was based on the patients’ symptoms and medical history, and was supported with laboratory findings. Both the modified Asthma Predictive Index (mAPI) and Global Initiative for Asthma (GINA) report 2014 were used [15, 16]. The patients, who had recurrent episodes (≥4) of wheezing, that responded to inhaled steroids and/or a bronchodilator taken within 2–4 weeks were diagnosed with reactive airway disease.

#### Protracted bacterial bronchitis (PBB)

The diagnosis was based on the presence of a chronic, wet cough, having the appropriate response to the antibiotic therapy and resolution of the cough within 2–4 weeks. No other alternative causes of this specific cough were found [1]. The FFB was also performed to exclude any other causes and to detect specific bacterial etiologies and neutrophilia in the BALF.

#### Upper airway cough syndrome (UACS)

The diagnosis was based on the patients’ medical history, the presence of postnasal discharge, nasal mucosal edema, hyperemia, and faintness, and the ability to respond to an antihistamine, nasal saline, and/or nasal steroid therapy in 2–4 weeks. Allergic rhinitis, and postnasal drip syndrome were included [5, 17]. Also, the diagnosis was supported with the evaluation of an otorhinolaringologist.

#### Gastroesophageal reflux disease (GERD)

The patients were diagnosed by a pediatric gastroenterelogist via esophageal Ph/impedence monitoring and a gastroduodenoscopy, which responded to the treatment of a proton pump inhibitor within 2 to 4 weeks.

#### Non-cystic fibrosis bronchiectasis

The diagnosis was affirmed with a thorax HRCT in the patients who had a persistent wet cough, and abnormal CXR findings, which did not respond to antibiotic therapy and also maintained non-regressive symptoms for 2–4 weeks [18].

#### Cystic fibrosis

The diagnosis was established with the presence of phenotypic characteristics, two positive measurements of sweat tests, and positive genetic mutation analysis.

#### Bronchiolitis obliterans (BO)

The diagnosis was based on the following criteria: a) patients with no other respiratory disease from birth to the onset of the acute disease such as bronchiolitis or severe pneumonia, c) patients with persistent obstructive respiratory symptoms for at least 60 days after the initial illness, d) patients with HRCT findings in a mosaic pattern, vascular attenuation, and expiratory air trapping, and e) excluding other diagnoses [19].

#### Tuberculosis

The diagnosis was usually based on persistent symptoms unresponsive to antibiotherapy, chest radiography findings, tuberculin skin test positivity, positive microbiologic tests, and history of contact with people having tuberculosis disease.

#### Psychogenic cough

The diagnosis was based on clinical symptoms such as bizarre honking coughs, which is very disruptive to daily life. The child looks healthy and the cough reduces to some extent when the child is sleeping [9].

#### Spontaneous remission

Improvement of cough during follow-up without treatment.

### Statistical analyses

SPSS 15.0 version was used for analysis. The numerical parameters were described with the mean, median, and standard deviation; distributions of the categorical measurements were determined by frequencies and percentages.

## Results

**Fig. 1:**
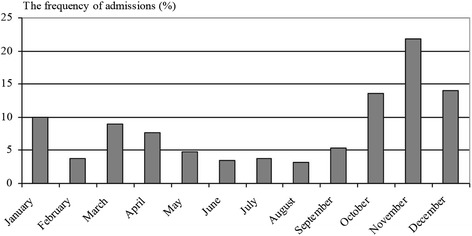
The frequency of admissions according to the months

In total, 563 patients were included in the study. The demographic and clinical characteristics may seen in Table 2. The most frequent hospital admissions were seen in November (123 (21.8 %) (Fig. 1).

**Table 2:** The demographic and clinical characteristics of the patients

Characteristics		Patients (*n* = 563)
Age, mean ± SD yr (min-max, mo)		5.4 ± 3.8 (2–204)
Age distribution n(%)	0–2 years	128 (22.7)
	2–6 years	240 (42.6)
	>6 years	195 (34.6)
Gender (female/male)		272/291
Cough duration at enrollment, mean ± SD mo		2.76 ± 2.69
Cough score at enrollment, mean ± SD		2.6 ± 1.2
Wet cough, n(%)		319 (56.7)
Household tobacco smoke, n (%)		162 (28.2)
Family history of atopy, n (%)		155 (27.7)

Of the 563 patients, 393 (69.8 %) children had a specific cough and 170 (30.2 %) had a non-spesific cough. The final diagnosis of the patients and also the final diagnosis according to the age groups were seen in Table 3.

**Table 3:** The last diagnosis of the patients

Last Diagnosis	n (%)			
	Total (*n* = 563)	0–2 years (*n* = 128)	2–6 years (*n* = 240)	>6 years (*n* = 195)
Atopic asthma	140 (24.9)	25 (19.5)	69 (28.8)	46 (23.6)
Reactive airway disease (Asthma-like symptoms)	107 (19.0)	38 (29.7)	52 (21.7)	17 (8.7)
Procracted bacterial bronchitis	67 (11.9)	11 (8.6)	33 (13.8)	23 (11.8)
Upper airway cough syndrome	51 (9.1)	6 (4.7)	26 (10.8)	19 (9.7)
Physicogenic cough	31 (5.5)	-	4 (1.7)	27 (13.8)
Non-cyctic fibrosis bronchiectasis	28 (5)	3 (2.3)	4 (1.7)	21 (10.8)
Bronchiolitis obliterans	28 (5)	10 (7.8)	13 (5.4)	5 (2.6)
Rhinosinusitis	26 (4.6)	2 (1.6)	16 (6.7)	8 (4.1)
Cystic fibrosis	20 (3.6)	11 (8.6)	1 (0.4)	8 (4.1)
Tuberculosis	19 (3.4)	2 (1.6)	4 (1.7)	13 (6.7)
Pneumoia-Bronchopneumonia	18 (3.2)	4 (3.1)	10 (4.2)	4 (2.1)
Gastro-esophageal reflux	15 (2.7)	9 (7)	5 (2.1)	1 (0.5)
Tracheo-bronchomalacia	5 (0.9)	4 (3.1)	-	1 (0.5)
Foreign body aspiration	3 (0.5)	2 (1.6)	1 (0.4)	-
Spontan resolution	2 (0.4)	-	1 (0.4)	1 (0.5)
Vasculer ring	1 (0.2)	-	-	1 (0.5)
Pulmonary hemosiderosis	1 (0.2)	1 (0.8)	-	-
Tumor (Ganglioneuroma)	1 (0.2)	-	1 (0.4)	-

### The results for specific cough

A total of 393 (69.82 %) patients with specific cough pointers (Table 1), an asthma diagnosis was made for the 140 (24.9 %) of them based on mAPI and GINA recommendations. Reactive airway diseases were diagnosed in the 43 (7.6 %) patients, with specific signs and symptoms.

A nasal steroid, a nasal salin solution, and an oral antihistamine were given to 87 (15.4 %) patients, where UACS was thought to be present. These patients were re-evaluated after 2–4 weeks and cough was found to be resolved in 51 (9.1 %), yet still persisting in 36 patients. The otorhinolaringologist evaluated the patients. Twenty-six of these patients (4.6 %) were diagnosed with rhinosinusitis and their cough was resolved after an oral amoxicillin-clavulanate therapy (40–60 mg/kg/day for 10 days). The rest of the 10 patients, who had especially dry cough, and were observed as having a non-spesific cough were consequently diagnosed with reactive airway disease. The inhaled steroids were taken and after 2–4 weeks, the cough was completely resolved in all patients.

In 47 (8.3 %) patients, reflux was suspected. After exposed symptoms were noted and clinical examinations were administered, they were referred to a pediatric gastroenterologist. After further tests, 15 (2.7 %) patients tested positive for reflux. After therapy with a proton pump inhibitor was administered, improvement was seen in all patients.

The other 118 (21 %) patients with spesific cough pointers had a productive cough, CXR abnormalities, and other clinical findings. The last diagnoses were non-cystic fibrosis bronchiectasis, BO, CF, tuberculosis, pneumonia and bronchopneumonia, foreign body aspiration, pulmonary hemosiderosis, ganglioneuromas (Table 3).

### The results for non-specific cough

**Fig. 2:**
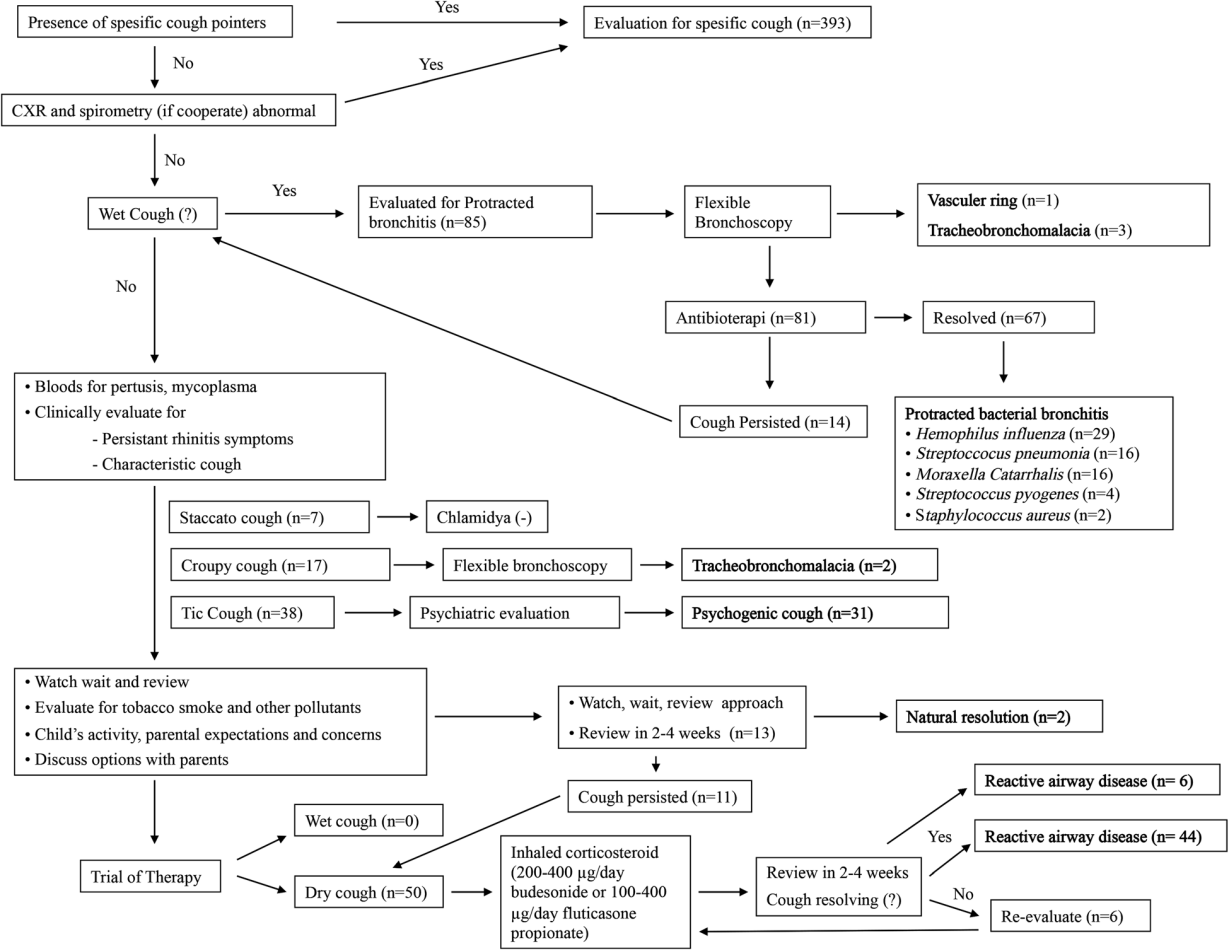
The evaluation of non-spesific cough

The patients who had no specific cough pointers were evaluated with the non-specific cough pathway. These patients had no spesific CXR abnormalities or spirometry findings. Eigthy-five (15.1 %) of the patients had a wet cough and 85 (15.1 %) of them had a dry cough. The evaluation of the patients with a non-specific cough can be seen in Fig. 2.

The patients with a wet cough were thought to be PBB. The flexible bronchoscopy was performed on these patients (*n* = 85). One patient had vascular ring, and 3 patients had tracheo-bronchomalacia with excessive and clear secretions. Additionally, their BALF culture results were negative. The other 81 patients had normal FFB findings. The BALF was taken from each patient and microbiological culture and neutrophilia were positive in 67 patients. The bacterial culture findings were 29 (43.2 %) *Hemophilus influenzae*, 16 (23.9 %) *Streptococcus pneumoniae*, 16 (23.9 %) *Moraxella Catarrhalis,* 4 (6 %) *Streptococcus pyogenes,* 2 (3 %) *Staphylococcus aureus.* The diagnosis of PBB was established in these 67 patients. The antibiotherapy (amoxicillin-clavulanate 60–80 mg/kg/day) was given and after 2–4 weeks, the cough was resolved in all patients.

The other 14 patients, without a positive BALF culture were also advised antibiotherapy. After 2–4 weeks, the cough persisted, but the characteristic of the cough changed into a dry cough, which was experienced especially at nights. An inhaled corticosteroid (200–400 μg/day budesonide or 100–400 μg/day fluticasone propionate) was given to these patients and their cough was resolved. These patients were diagnosed with reactive airway disease.

The patients with a dry cough were evaluated again (Fig. 2). The blood test results were negative for mycoplasma and pertusis. They didn’t have any symptoms suggesting allergic rhinitis. A characteristic cough was seen in 45 of these patients. 17 had croup cough, 7 had staccato cough, and in 38 patients, there was no cough experienced at nights. The pediatric psychiatrist evaluated these 38 patients, 31 of them were diagnosed with psychogenic cough. Chlamydia and Mycoplasma antibodies were found negative in the 7 patients, who had the staccato cough. The patients, who had croup cough, underwent FFB and 2 patients had tracheo-bronchomalacia.

The rest of the patients (*n* = 52) and their families were informed about the conditions of persistant cough and other follow-up optsions. We did not administer any therapy to 13 patients. After 2–3 weeks, spontaneous resolution was seen in 2 patients. Inhaled corticosteroids (200–400 μg/day budesonide or 100–400 μg/day fluticasone propionate) were administered to 50 patients. After 2–4 weeks, 6 patients had shown significant improvement, and 44 patients had no more cough at all. These six patients were educated about inhalation techniques, and the therapy continued. The cough was resolved after 4 weeks and we also diagnosed these patients with reactive airway disease. The total number of reactive airway disease in dry cough were 64 (11.4 %).

## Discussion

In this study, we found that the most common cause of chronic cough in children was asthma and asthma-like symptoms. The order of other common causes changed according to age groups. PBB was the second most common cause in children under 6 years of age, while the psychogenic cough was the second cause of cough in children over 6 years of age. To the best of our knowledge, the current study had the largest study population, which included pediatric patients in the literature, giving the frequency of diagnosis according to the ages.

The chronic cough is a common presenting symptom, and the evaluation of children with chronic cough still contains a major challenge for physicians due to the scarcity of data. Chang et al. highlighted that their own experience, and international data reflect the need to improve the management of chronic cough in children [7]. They made an evidence-based clinical management pathway and showed that this pathway has feasible, reliable, and improved clinical outcomes [1, 7]. Chang et al. mentioned that many cough pathways or guidelines were used, but none has been subjected to a randomized study. Also, they said taht this clinical algorithm has the potential to reduce the morbidity of chronic cough, unnecessary costs, and adverse events associated with mediacation use (7). Except Chang et al. [1], this is the first study, using these algorithm in evaluating children with the chronic cough. Additionally, we saw that this algorithm was advantageous, simple, and could also be easily used by primary physicians.

In several studies, different order of etiologies was given in children with the chronic cough [1, 20–23]. Asthma and asthma-like symptoms were found as the most common causes in some studies [22, 23], while PBB was detected the most common one in others [1, 20]. The differences in the causes of the final diagnosis may be due to the different referral practices from primary care, the different ages of children, and the prevelance of different local diseases [9].

Marchant et al. (median age 2.6-years) and Chang et al. (mean age 4.5 ± 3.7-years) reported the most common cause of chronic cough in children, in Australian settings, differed from those commonly reported in other childhood studies [1, 20]. They found the most common cause (40 and 41.6 %, respectively) as PBB. The etiologies to be expected are more than others, as asthma, GERD, and UACS were accounted for in only 10 %. These studies were performed in tertiary clinics. The other common causes may be diagnosed and treated in primary and secondary health care centers, which can result in a lower number of admissions, or referrals of patients with common causes to these tertiary clinics. Our hospital provides services to both secondary patients from pediatricians, and tertiary patients from other specialists. We took-in both secondary and tertiary patients to reflect the optimum general pediatric population. Asthma and asthma-like symptoms were the most common causes in our study.

The different ages of children studied may play an important role in the causes of chronic cough. In addition to the differences in practice settings, the etiologies and burden of chronic cough are also potentially influenced by age and studies published about these influences are scarce [8]. Asilsoy et al. (mean age 8.44 ± 2.13 years) found the most common cause as asthma as in the current study and also suggested that the reason for different results from Marchant et al. and Chang et al. study was due to the younger age of the study population [22]. Our study revealed that after 6-years of age, asthma was the first cause, but the second cause was psychogenic cough. As in our study, the proportion of etiologies can be changed according to the different age groups in children [10].

One of the reasons for different results among the studies may be due to the prevelance of various local diseases [9]. Supporting this idea, Turkish studies show a high proportion of asthma [22, 23], when compared with American and Australian studies [1, 20, 23]. Contradicting this idea, Asilsoy et al. [22] and found the PBB frequency in patients is very similiar with Marchant et al. [20], Chang et al. [1], while Koshoo et al. [21] and Karabel et al. [23] did not report a significant amount of PBB frequency.

The other less common etiologies such as bronchiectasis, cystic fibrosis, BO, tuberculosis, tracheo-bronchomalacia, foreign body aspiration, spontan resolution, vasculer ring, pulmonary hemosiderosis and tumor were also not common in current study, as in the literature. In contrast to our study, GERD was highlighted as a common etiology for chronic cough in some studies [21, 23]. Khoshoo et al. found it as a single most common factor associated with chronic cough by itself [21]. However, the relation between cough and GERD in children has not been disclosed completely [24]. When compared with other studies, a psyhogenic cough was detected more commonly in our study, especially in children over 6-years of age. Asilsoy et al. and Karabel et al. did not find any patients with this diagnosis [22, 23] while Chang et al. and Marchant et al. [1, 20] found 4.9 and 1 %, respectively.

## Conclusion

In our study, asthma and asthma-like symptoms were the most common diagnosis in children. Different age groups in children may have a different order of frequencies. Psychogenic cough should be thought of in the common causes especially in older children.

## Abbreviations

CF: Cystic fibrosis
CXR: Chest X-ray
PFT: Pulmonary function test
Ig: Immunglobulin
HRCT: High resolution computarized tomography
FFB: Flexible fiberoptic bronchoscopy
BALF: Bronchoalveolar lavage fluid
mAPI: Modified asthma predictive index
GINA: Global Initiative for Asthma
PPB: Protracted bacterial bronchitis
UACS: Upper airway cough syndrome
GERD: Gastroesophageal reflux disease
BO: Bronchiolitis obliterans
